# S-leaping: an efficient downsampling method for large high-throughput sequencing data

**DOI:** 10.1093/bioinformatics/btad399

**Published:** 2023-06-24

**Authors:** Hiroyuki Kuwahara, Xin Gao

**Affiliations:** Computer, Electrical and Mathematical Sciences and Engineering Division (CEMSE), Computational Bioscience Research Center (CBRC), King Abdullah University of Science and Technology (KAUST), Thuwal 23955-6900, Saudi Arabia; Computer, Electrical and Mathematical Sciences and Engineering Division (CEMSE), Computational Bioscience Research Center (CBRC), King Abdullah University of Science and Technology (KAUST), Thuwal 23955-6900, Saudi Arabia

## Abstract

**Motivation:**

Sequencing coverage is among key determinants considered in the design of omics studies. To help estimate cost-effective sequencing coverage for specific downstream analysis, downsampling, a technique to sample subsets of reads with a specific size, is routinely used. However, as the size of sequencing becomes larger and larger, downsampling becomes computationally challenging.

**Results:**

Here, we developed an approximate downsampling method called s-leaping that was designed to efficiently and accurately process large-size data. We compared the performance of s-leaping with state-of-the-art downsampling methods in a range of practical omics-study downsampling settings and found s-leaping to be up to 39% faster than the second-fastest method, with comparable accuracy to the exact downsampling methods. To apply s-leaping on FASTQ data, we developed a light-weight tool called fadso in C. Using whole-genome sequencing data with 208 million reads, we compared fadso’s performance with that of a commonly used FASTQ tool with the same downsampling feature and found fadso to be up to 12% faster with 21% lower memory usage, suggesting fadso to have up to 40% higher throughput in a parallel computing setting.

**Availability and implementation:**

The C source code for s-leaping, as well as the fadso package is freely available at https://github.com/hkuwahara/sleaping.

## 1 Introduction

Downsampling is a valuable technique that is routinely used to evaluate the design of high-throughput sequencing (HTS) studies. Be it whole-genome sequencing (WGS), RNA sequencing, or DNA methylation sequencing, researchers can use downsampling to conveniently and economically sample HTS data with various sizes. Among recent use cases of downsampling is low-coverage WGS (lcWGS), an emergent alternative to SNP genotyping arrays in population genetics, thanks to a steady decrease in sequencing costs. Indeed, because downsampling can efficiently generate lcWGS data from WGS data, it has been used to evaluate the performance of genotype imputation methods—inference methods that increase genotype density by predicting unobserved genotypes (Gilly *et al.* 2019, Li *et al.* 2021, Lou *et al.* 2021, Rubinacci *et al.* 2021)—and to determine cost-effective sequencing coverage rates for a range of computational medicine applications (Goldfeder *et al.* 2016, Homburger *et al.* 2019, Davies *et al.* 2021, Sun *et al.* 2021).

There are a number of downsampling tools available for HTS data, but with different specifications. For example, the downsampling features in picard, GATK (McKenna *et al.* 2010), and SAMtools (Li *et al.* 2009) select reads at an equal probability. Although this type of downsampling is computationally efficient with a very small memory footprint, the user cannot ensure that the obtained data have a desired number of reads. Another type of downsampling is reservoir sampling, which makes a single pass through an HTS file and unbiasedly samples the specific number of reads without having to know the total number of reads. This downsampling approach is used in seqtk (https://github.com/lh3/seqtk), a most commonly used tool to downsample FASTQ data. Because reservoir sampling allows sampling of exactly *k* elements from a population of unknown size *n* in a single pass, this article focuses on this type of downsampling.

Numerous algorithms have been proposed for reservoir sampling. Waterman developed the direct approach called Algorithm R (Knuth 1997) that scans and processes each element for random selection. Although this algorithm is simple, it demands the generation of one unit uniform random variate per element, which becomes inefficient when selecting small subsets of the data. To deal with such cases, a different type of reservoir sampling was developed to focus on the number of steps it takes to select the next element, rather than processing every single element (Vitter 1985, Li 1994). To process large HTS datasets, with hundreds of millions, or even billions, of reads per sample, however, the use of the existing reservoir-sampling methods is found to be inefficient, rendering the development of a more computationally efficient method not only essential but also necessary.

Here, we developed s-leaping, a method that focuses on downsampling of large datasets by approximating reservoir sampling. By applying the concept of leaping to downsampling, s-leaping simplifies the sampling procedure and reduces the average number of random numbers it requires. Comparing s-leaping’s performance with that of several exact reservoir-sampling methods for a task of selecting 10–40 million elements from a population of 100–500 million, we found s-leaping to be up to 39% faster than the most efficient exact method, while showing no sign of deteriorating accuracy. We implemented s-leaping in a tool called fadso that specializes in downsampling of FASTQ data, compared its efficiency against that of seqtk, and found fadso to be more efficient in terms of computation time and memory usage than seqtk.

## 2 Results

### 2.1 Theoretical evaluation of reservoir-sampling methods

We define reservoir sampling as a task to randomly and unbiasedly choose *k* elements from a file holding elements of unknown size *n* in a single pass. In Algorithm R, the reservoir initially comprises the first *k* elements, and for all i>k, the *i-*th element is selected at probability k/i to replace a randomly selected element in the reservoir. At the end of *j-*th step (j≥i), Algorithm R ensures that each element in the reservoir has an equal probability (i.e. k/j) of being selected and kept in the reservoir (Knuth 1997). Another type of reservoir sampling focuses on the number of steps it takes to select the next element, which we call next selection method. A next selection method randomly makes two decisions per selected element: the step size to the next selection and the index of an element to be replaced in the reservoir.

Both approaches have pros and cons. Computation-wise, the selection operation is simpler in Algorithm R than the next selection method. Indeed, direct sampling of step size in the next selection method is complicated and existing algorithms often use rejection sampling with easier envelope functions (Vitter 1985, Li 1994). Selection-wise, after processing the first *k* elements, Algorithm R requires one random number per element, whereas the next selection method demands at least two random numbers per selection. However, when the probability of selection is small (i.e. k/i≪1), the next selection method can skip many elements, whereas Algorithm R still needs to process every single element. To process *n* elements, Algorithm R requires n−k random numbers, whereas the optimum next selection method is expected to need approximately 2kln(n/k). We estimated an optimal next selection method to demand fewer random numbers than Algorithm R when k<0.3n.

### 2.2 Overview of s-leaping

S-leaping is a hybrid method that combines Algorithm R and an efficient approximate next selection method. It follows Algorithm R for the first 2k-th elements—when the probability of selecting each element is at least 0.5 (i.e. k/i≥0.5). From the (2k+1)-th element onward, it devises an approximate next selection that simplifies the sampling of the next step size by introducing a leaping interval, *s*.

To sample the step size in s-leaping, we assume that the selection probability does not change within *s*: for all i∈[m,m+s) where m≥2k+1, the probability of selecting the *i-*th element is approximated by k/(m+0.5s). This assumption allows us to simplify the step-size distribution substantially and sample the step size from a geometric distribution with p=k/(m+0.5s). This simplified distribution approximates the true step-size distribution well when the lower bound of *m* (i.e. 2k+1) is much larger than *s*. In the case of downsampling for HTS data, which easily comprise hundreds of millions of reads, we assume this condition to be met safely even when the leap size is in the order of thousands.

### 2.3 Optimization of the number of random numbers

In s-leaping, the use of Algorithm R to process the first 2k elements serves two objectives: to increase the step-size sampling accuracy by making m≥2k, as described in the previous section, and to reduce the number of random numbers needed in the method. For the first 2k elements, s-leaping requires *k* random numbers; for the rest of the elements, approximately 2kln[n/(2k)] random numbers on average. After algebraic manipulations, these make 2kln(n/k)−[2ln(2)−1]k in total, implying that s-leaping requires approximately 0.39k fewer random numbers than the optimum next selection method.

To compare s-leaping with Algorithm R in terms of the required number of random numbers, let us first consider the selection after the 2*k*-th element. Because the step size is sampled from a geometric distribution with *p* < .5, s-leaping is expected to generate step sizes larger than two. Although s-leaping requires two random numbers per selection, this expected step size implies that s-leaping requires on average fewer than one random number per element after the 2*k*-th element, which in turn implies that s-leaping is expected to require fewer random numbers than Algorithm R when n>2k. Because s-leaping is guaranteed to have the same number of random numbers as Algorithm R for all n≤2k, the number of random numbers that s-leaping demands is always fewer than or equal to that of Algorithm R.

**Figure 1.**
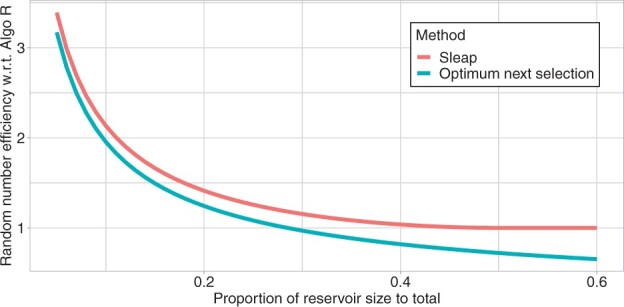
A theoretical comparison of estimates of random numbers required for each method. The *x*-axis is the proportion of the reservoir size to the total size; the *y*-axis is the ratio of an estimated number of random numbers needed in each method to the number of random numbers required in Algorithm R.

To analyze how the relative size of *k* with respect to *n* affects the number of required random numbers in each method, we expressed as functions of a proportional size factor the ratio of the random numbers required in s-leaping and the optimal next selection method over the random numbers required in Algorithm R (Fig. 1). This shows that the efficiency of the optimum next selection method decreases more drastically than that of s-leaping as the reservoir-size proportional factor increases: when k=0.1n, s-leaping is 110% more efficient and the optimum next selection method is 100% more efficient than Algorithm R; when k=0.3n, s-leaping, 15% more efficient, the optimum next selection method, 3% less efficient; and when k=0.4n, s-leaping, 4% more efficient, the optimum next selection method, 18% less efficient.

### 2.4 Accuracy evaluation of s-leaping

As a proof-of-concept, we implemented s-leaping, along with three downsampling methods, Algorithms R, L, and Z (Vitter 1985, Li 1994, Knuth 1997), in C. Algorithms L and Z are both next selection methods, but with widely different algorithmic designs for step-size sampling. Algorithm L requires three random numbers per selection, but with a simpler step-size sampling procedure; Algorithm Z requires two random numbers per selection, but with a more complex step-size sampling procedure.

In this experiment, we chose *n* to be 100, 300, or 500 million, while varying *k* to have four values: 10, 20, 30, and 40 million. (Note that although the value of *n* was decided in prior, this information was used only for the performance evaluation purpose only.) These numbers are within the realm of biological relevance: with 300 bp read-pair length, these numbers roughly correspond to downsampling from 9×, 27×, and 45× coverage data to 0.9×, 1.8×, 2.7×, and 3.6× coverage data in the human genome. These settings also allowed us to evaluate the performance in a reasonable range of k/n—from 0.02 to 0.4. For each combination of *k* and *n*, we ran each method 10 times with different random seeds. In s-leaping, we fixed the leap-size factor to be 0.005 (i.e. s=0.005k). Because it devises an approximated next-selection method, s-leaping, along with Algorithms L and Z, is referred to as a next selection-based method.

**Figure 2.**
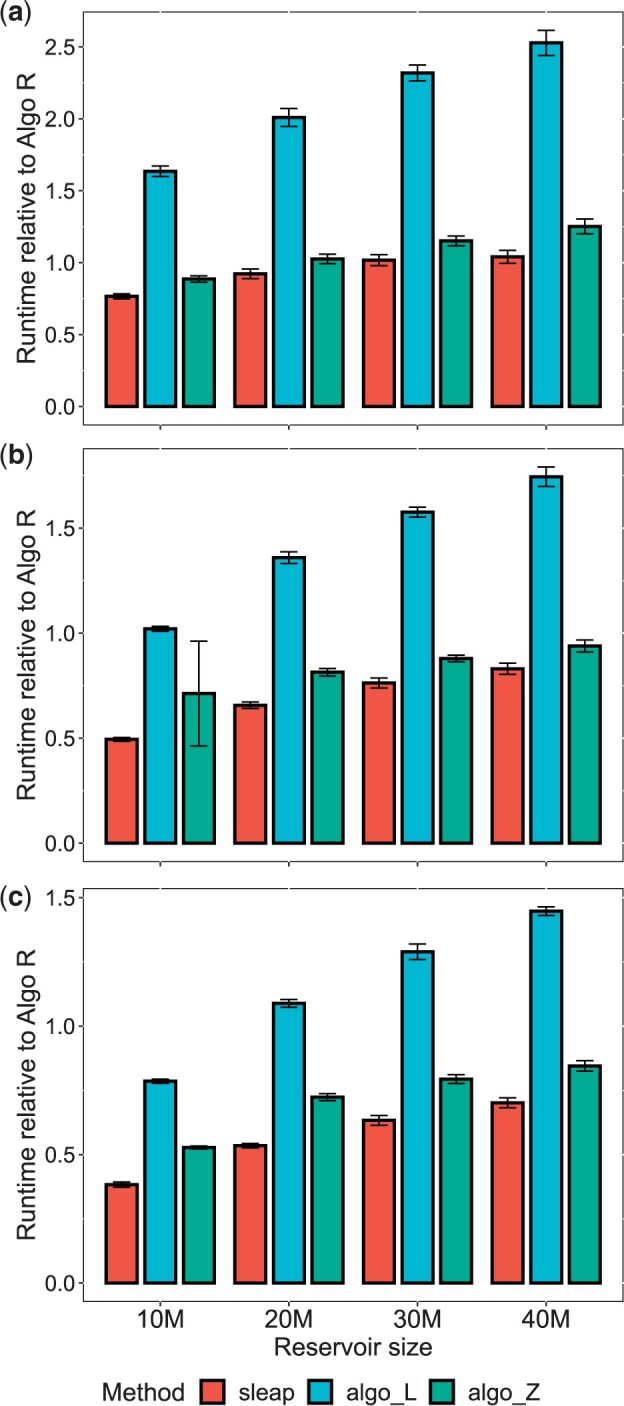
A comparison of runtime among the four downsampling methods. The *x*-axis is the reservoir size; the *y*-axis is the average runtime relative to that of Algorithm R. Error bars indicate 95% confidence level by a second-order Taylor approximation. (a) The runtime results from downsampling of 100 million elements; (b) 300 million; (c) 500 million. Here, leap indicates s-leaping; algo_R, Algorithm R; algo_L, Algorithm L; algo_Z, Algorithm Z.

First, we analyzed how efficient the three next selection-based methods were in comparison with Algorithm R. To this end, we computed the ratio of the average runtime of each next selection-based method to that of Algorithm R. In this setup, thus, a runtime ratio less than 1 indicates that a next selection-based method has better computational efficiency than Algorithm R. As expected, all the next selection-based methods had a trend to increase efficiency as the k/n ratio decreased (Fig. 2); however, they had widely different conditions under which to outperform Algorithm R. S-leaping was more efficient than Algorithm R when k/n≤0.2 and they were comparable when k/n is 0.3 [1.02, 95% confidence interval (CI): 0.98–1.06] and 0.4 (1.04, 95% CI: 1.00–1.09). Algorithm Z was more efficient than Algorithm R when ≤0.133, and they were comparable when k/n is 0.2 [1.03, 95% CI: 0.99–1.06], whereas Algorithm L was more efficient than Algorithm R only when ≤0.02. Among the three next selection-based methods, s-leaping was consistently the fastest, outperforming Algorithm R in most of our *k*−*n* settings; Algorithm Z, the second; Algorithm L, the distant third. Although s-leaping and Algorithm Z both showed strong competitive advantages over Algorithm R when the k/n ratio was smaller, s-leaping demonstrated higher efficiency than Algorithm Z. For example, when n=500 million, s-leaping was 20% faster than Algorithm Z with k=40 million (s-leaping, 0.70, 95% CI: 0.68–0.72; Algorithm Z, 0.85, 95% CI: 0.82–0.87) and it was 39% faster with k=10 million (s-leaping, 0.38, 95% CI: 0.37–0.39; Algorithm Z, 0.53, 95% CI: 0.52–0.53).

### 2.5 Accuracy evaluation of s-leaping

Next, because s-leaping is an approximated downsampling method, we evaluated its ability to generate unbiased samples. To this end, we used the maximum D—the maximum discrepancy between the cumulative distributions of two samples that the Kolmogorov–Smirnov test uses as the test statistic. We measured the maximum D between the selected *k* elements and the unit uniform distribution, allowing us to evaluate the degree of uniformity in the selected elements. We computed the maximum D for each downsampling run from the experiment described in the previous section and measured the maximum D distribution for each method.

**Figure 3.**
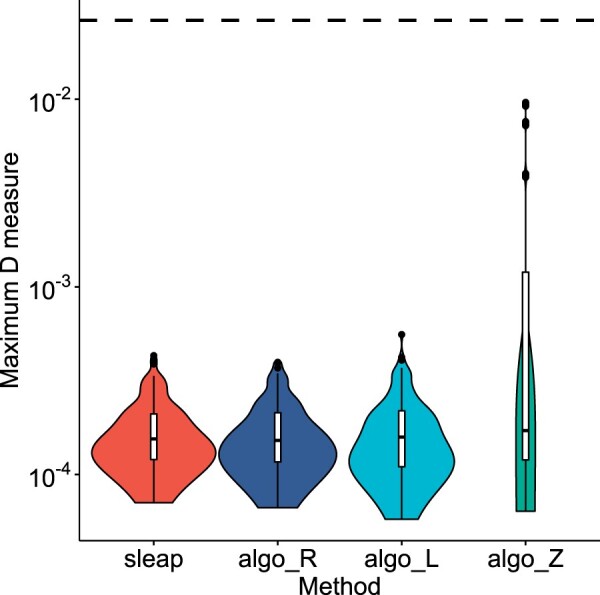
Results from analysis of goodness-of-fit to uniform distribution. The *x*-axis is the four downsampling methods; the *y*-axis is the maximum D measure against the uniform distribution. A violin plot shows the distribution of maximum D samples from a downsampling method. The dashed horizontal line indicates the threshold level of the Kolmogorov–Smirnov test at α=0.999. Here, sleap indicates s-leaping; algo_R, Algorithm R; algo_L, Algorithm L; algo_Z, Algorithm Z.

We found that the maximum D values from s-leaping (mean $1.75\times 10^{-4}$, sd $7.46\times 10^{-5}$) were distributed similarly to those from Algorithms R (mean $1.72\times 10^{-4}$, sd $7.49\times 10^{-5}$) and L (mean $1.78\times 10^{-4}$, sd $8.78\times 10^{-5}$), showing no evidence of s-leaping with lower accuracy than those methods (Fig. 3). In fact, s-leaping had more consistent and lower maximum D measures than those from Algorithm Z (mean $1.84\times 10^{-3}$, sd $3.15\times 10^{-3}$), suggesting that s-leaping had a better goodness-of-fit than Algorithm Z. To further evaluate the accuracy of s-leaping (i.e. the goodness of fit to a uniform distribution), we computed the critical value at the significance level α=0.999—the value at which the probability to reject the null hypothesis is 0.999 in the Kolmogorov–Smirnov test—and compared the maximum D values with this critical value. The critical value was found to be $2.63\times 10^{-2}$; this value was substantially higher than all the maximum D values from s-leaping, along with the other methods (Fig. 3).

We have thus far set the leap size *s* to be proportional to *k* with a fixed proportion, s=0.005k (e.g. when k= 10 million, s= 50 000). Our results indicated that this leap-size proportional factor was small enough to have accuracy comparable to that of the exact reservoir-sampling methods. To further evaluate the accuracy of s-leaping, we analyzed the extent to which the accuracy of s-leaping changes with respect to the leap-size factor. To this end, we set k= 20 million and *n* to be 100, 300, or 500 million, we ran s-leaping 10 times for each setup, and computed maximum D for each leap-size factor value. We set the leap-size factor to take nine values ranging from 0.001 to 0.5 (i.e. *s* ranges from 20 000 to 1 million).

**Figure 4.**
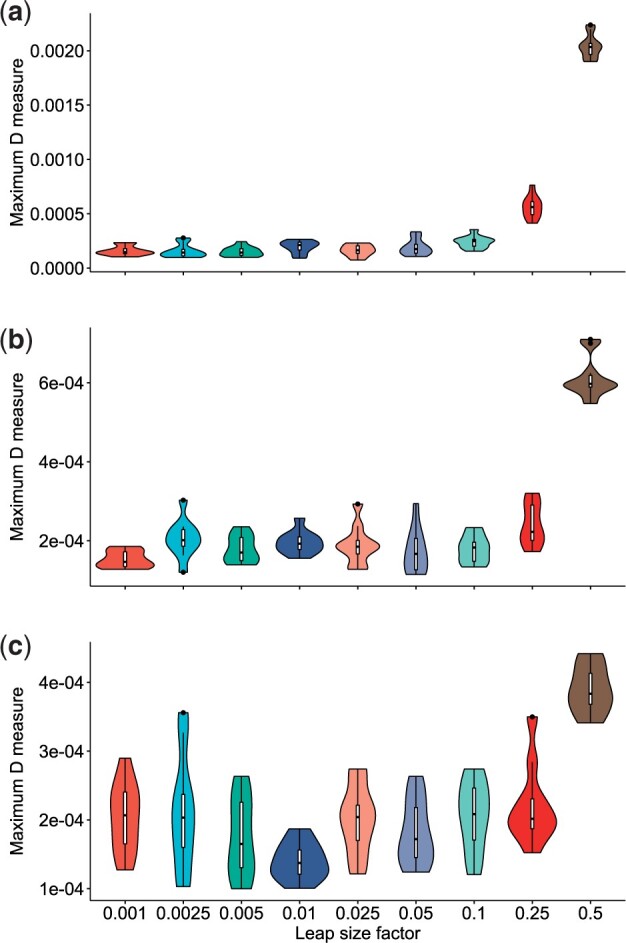
Comparison of downsampling accuracy with respect to different leap size factor values. The maximum D distributions of nine different leap size factor values are compared. The reservoir size *k* is set to 20 million, while the population size *n* is changed to have three different values: (a) *n* = 100 million; (b) 300 million; and (c) 500 million. The *x*-axis is the leap-size factor; the *y*-axis is the maximum D measure against the uniform distribution. Each distribution is generated from 10 samples.

We had expected maximum D to be an increasing function of leap-size factor and a decreasing function of *n*—i.e. the accuracy of s-leaping becomes worse with a larger leap size and smaller total size. Indeed, we observed this pattern, but only to a limited extent: maximum D showed clear positive relation patterns only for large leap-size factor values (from 0.1 to 0.5) with n= 100 and 300 million (Fig. 4). Regardless of the value of *n*, we found maximum D to have similarly small values below $4.0\times 10^{-4}$ for leap-size factor ranging from 0.001 to 0.1, indicating that within this range, the accuracy of s-leaping is high and independent of the leap size. For example, when the leap-size factor is 0.1 (i.e. s=200,000), the mean maximum D was $2.39\times 10^{-4}$ (sd $5.63\times 10^{-5}$) with *n* = 100 million; $1.79\times 10^{-4}$ (sd $3.49\times 10^{-5}$) with *n* = 300 million; and $2.06\times 10^{-4}$ (sd $5.08\times 10^{-5}$) with *n* = 500 million. Under all the settings we studied, we found the maximum D values to be well below the critical value at significance level α=0.999, demonstrating high levels of accuracy in a wide range of conditions.

### 2.6 Fadso: an implementation of s-leaping for FASTQ downsampling

To apply s-leaping on FASTQ data, we have developed fadso (FAstq DownSampling Optimizer), a command-line tool that downsamples FASTQ data to have the user-specified read size. This tool takes either gzipped or plain FASTQ files, downsamples single- or paired-end reads, and implements s-leaping, along with Algorithm L (Li 1994), Algorithm R (Knuth 1997), and Algorithm Z (Vitter 1985).

To evaluate the performance of fadso, we compared its speed and memory usage with those of seqtk, an efficient and lightweight tool for processing sequencing data in the FASTQ format, with a downsampling feature to output a desired number of reads. To this end, we selected s-leaping as the downsampler in fadso. Through source-code inspection, we found that seqtk implemented Algorithm R for downsampling of FASTQ data with a specific target size. Note that both fadso and seqtk are single-threaded programs written in C.

To measure the downsampling performance, we used the forward-read set of ERR174310 from the Illumina Platinum genome project (Eberle *et al.* 2017) that has 208 million 101 bp reads. We downsampled this read set to target sizes ranging from 5 to 20 million with the 5 million-size increment and repeated this procedure for five times with different random seeds. We evaluated the downsampling performance by measuring the runtime and peak memory usage of the programs.

**Figure 5.**
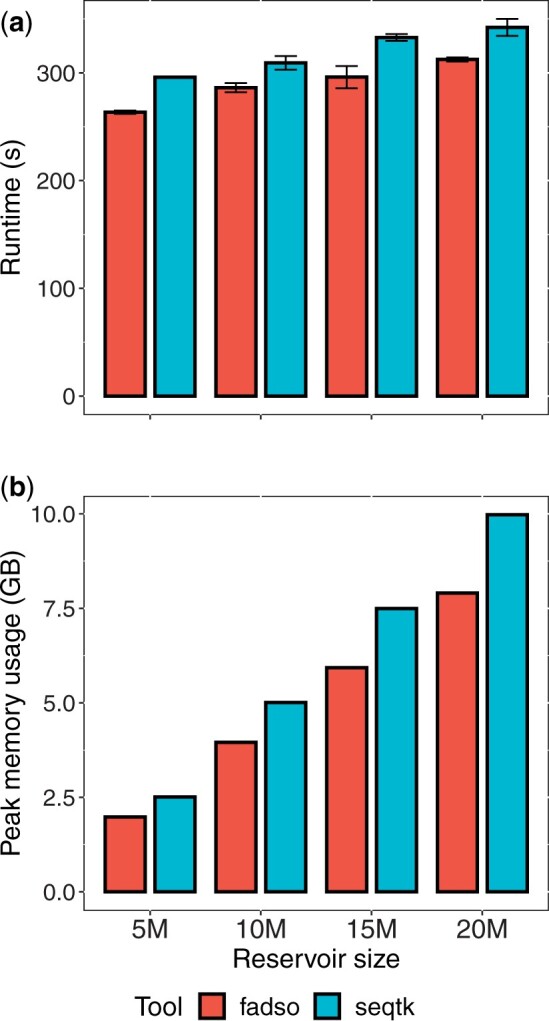
Performance comparison of FASTQ downsampling tools. The *x*-axis is the target read count; the *y*-axis is the performance measure (n=5 for each setting). (a) Runtime was measured in seconds; (b) peak memory usage, in gigabytes. Bars indicate sample average from five runs; the error bars, sample standard deviations.

Fadso consistently outperformed seqtk in terms of speed: fadso averaged 12% speedup for the 5 million downsampling size; 8% for the 10 million size; 12% for the 15 million size; and 10% for the 20 million size (Fig. 5a). The results from the peak memory usage showed fadso to consistently have smaller memory footprints than seqtk, reducing the memory requirement by around 21% in each downsampling size setting (Fig. 5b).

## 3 Discussion

Our results demonstrated s-leaping to be highly efficient, while achieving accurate downsampling—with no sign of deteriorating accuracy, comparable to exact reservoir-sampling methods. The high performance of s-leaping arose from the assumption that the selection probability changes negligibly within a relatively small interval compared with the reservoir size. Not only did this leaping assumption simplify the sampling of next selections, but it also accelerated the calculation of the selection probability. These improved the runtime efficiency especially when n≫k≫1.

This type of “leaping” strategies to accelerate the runtime by sacrificing the exactness is not new; indeed, in the field of stochastic chemical kinetics, an approximate simulation method called tau-leaping (Gillespie 2001) was shown to improve the time efficiency over an exact method called Gillespie’s stochastic simulation algorithm (Gillespie 1976)—which simulates every single reaction event in a well-stirred chemically reacting system governed by a continuous-time jump Markov process. By assuming reaction rates to change negligibly within a specified time interval, the tau-leaping method allows the system to leap along each interval. Although the basis of the speedup is different between s-leaping and tau-leaping, they both stem from the same underlying concept, which sacrifices miniature differences from the exact solution within subintervals to increase computational efficiency. Our study provided evidence for a successful application of a leaping strategy for an important task in omics research—downsampling of large HTS data.

To apply s-leaping to downsampling of FASTQ data, we developed fadso, a downsampling tool implementing s-leaping for FASTQ data. In terms of both runtime and memory usage, fadso was more efficient than seqtk, the leading FASTQ processing tool implementing Algorithm R to downsample FASTQ data. However, considering the performance results of s-leaping with respect to Algorithm R, we found fadso’s speedup improvement to be lower than expected. This can be explained by the computational overhead involved in reading, uncompressing, and parsing compressed FASTQ files that might have weakened the effects of the algorithmic speedup of s-leaping. Another possibility could be that the implementation of seqtk to parse compressed FASTQ files could be more efficient than that of fadso, which in turn might have overtaken some of s-leaping’s algorithmic advantage, suggesting that fadso could have some room to further increase its efficiency with a more efficient FASTQ parser in the future. Nevertheless, given fadso’s superior performance in memory usage, our results suggest the current version of fadso to process large FASTQ datasets at up to 40% higher throughput rate than seqtk in parallel computing settings.

## 4 Conclusion

Here, we introduced s-leaping, a novel downsampling algorithm tailored to downsample large data by approximating the next step-size probability using the concept of leaping, and developed fadso, a downsampling tool for FASTQ that implements s-leaping. We have shown that the accuracy of s-leaping is on par with exact reservoir-sampling methods and that the Fadso was deposited in Github. Its package is available to download at https://github.com/hkuwahara/sleaping.

## 5 Methods

### 5.1 Algorithm R

Reservoir sampling sequentially reads a streaming file and randomly samples *k* elements with the equal probability from the population of unknown size *n*. Waterman developed a direct approach of reservoir sampling called Algorithm R in the 1970s (Knuth 1997). Algorithm R first places the first *k* elements in the selection set called “reservoir” of size *k*, and for all i>k, it selects the *i-*th element at probability k/i to replace a randomly picked element in the reservoir. At the end of *j-*th step (j≥i), each element in the reservoir is guaranteed to have probability k/j in the reservoir. This algorithm is simple and requires only one random variate per element, requiring n−k random variates in total.

### 5.2 Next selection method

We define the next selection method as another type of reservoir sampling that focuses on the number of steps it takes to select the next element. Just like Algorithm R, the next selection method first fills the reservoir with the first *k* elements. For all i>k, it then randomly makes two decisions per selected element: the step size to the next selection and the index of an element in the reservoir to be replaced. Its sampling distribution can be derived from the probability to select the *i-*th element, k/i. Let us first define $p_{n}(\nu |i)$ to be the probability that it takes exactly ν steps to select the next element for the reservoir after the *i-*th element is processed. Then, we can express $p_{n}(\nu |i)$ as where p(i+j) is the probability of selecting the (i+j)-th element.


$$p_{n}(\nu |i)=p(i+\nu )\prod_{j=1}^{\nu -1}\left[1-p(i+j)\right]=\frac{k}{i+\nu }\prod_{j=1}^{\nu -1}\frac{i+j-k}{i+j},$$

Vitter developed Algorithm Z that faithfully samples ν using this distribution or using the rejection-acceptance sampling with an easier envelop (Vitter 1985). Li (1994) developed Algorithm L that treats the reservoir-sampling problem as a task of selecting indices of *k* smallest numbers from *n* randomly drawn numbers. Although the approach to select the next element can be widely different among different algorithms, the next selection method in general places the first *k* elements in the reservoir and for all i>k, selects on average $\sum_{i=k+1}^{n}k/i\approx k\left(\ln\left(n\right)-\ln\left(k\right)\right)=k\ln\left(n/k\right)$ elements at the *n-*th elements. The time complexity of the next selection method, including Algorithms L and Z, was thus reported to be O(k(1+ln(n/k))) (Vitter 1985, Li 1994).

### 5.3 Next selection sampling in s-leaping

To derive the step-size sampling used in s-leaping, we start from the definition of $p_{n}(\nu \;|\;i)$. Direct sampling of ν from $p_{n}(\nu \;|\;i)$ is complicated and s-leaping uses an approximation. To approximate $p_{n}(\nu \;|\;i)$, we first define a leap size *s* that is much smaller than the reservoir size (i.e. s≪k). Within this relatively small leap subinterval, we assume that differences in p(i+j) are insignificant and that for all i∈[m+1,m+s], p(i) is well approximated by k/(m+0.5s). This allows us to approximate $p_{n}(\nu |i)$ by the geometric distribution with p=k/(m+0.5s):


$$p_{1}(\nu \;|\;i)\approx \frac{k}{m+0.5s}{\left(1-\frac{k}{m+0.5s}\right)}^{\nu -1}.$$

Thus, with the inverse transform sampling, we can sample a geometric random variate ν as follows: where λ=ln(1−k/(m+cs)) and *u* is a unit uniform random variate. The sampling of ν requires only one unit uniform random variate, demanding only two unit uniform random variates per selection step. In addition, because the distribution of ν is fixed within each leap subinterval, the sampling of ν can be streamlined by reusing λ.


$$\nu =\left\lfloor \frac{\ln(u)}{\lambda }\right\rfloor +1,$$

### 5.4 S-leaping algorithm

Below shows the algorithm of s-leaping with zero-based numbering:

Require: *k* the reservoir size; ϵ the leap-size factor;

1: for *i* from 0 to 2k−1, select elements using Algorithm R;

2: set i←2k−1; s←ϵk;

3: **while** not end of file **do**

4:   set j←0; p←k/(i+1+0.5s); λ←ln(1−p);

5:   **while** True **do**

6:    set u← a unit uniform random variate; ν←⌊ln(u)/λ⌋+1;

7:    update j←j+ν;

8:    **if**j≥s**then**

9:     update i←i+s, and break from the loop;

10:    **end if**

11:    **if** end of file **then**

12:    terminate;

13:    **end if**

14:    set r← a discrete uniform random variate in [0,k−1];

15:    update the *r*-th reservoir element by the (i+j)-th element;

16:   **end while**

17: **end while**

The leap-size factor ϵ needs to be small in order to have reasonable accuracy. We used 0.005 as the default value of ϵ.

### 5.5 Estimation of the number of random numbers needed in the next selection method

First, we estimated the average number of selected elements after processing n−k elements in the next selection method, ignoring the first *k* elements that were used to fill the reservoir initially. Because the probability to select the *i*-th element is k/i for all i>k, the average number of selected elements can be expressed by a harmonic series and approximated using the natural logarithm:


(1) $$\sum_{i=k+1}^{n}\frac{k}{i}\approx k\int_{k+1}^{n}\frac{1}{x}dx=k\ln\left(\frac{n}{k+1}\right)\approx k\ln\left(\frac{n}{k}\right).$$

Let *t* be the average number of random numbers needed to select the next element per selection step. Then because each selected element needs to replace a randomly picked element in the reservoir, we can approximate the average number of random numbers needed in the next selection method as follows:


(2) $$k\left(t+1\right)\ln\left(\frac{n}{k}\right).$$

Because every step-size sampling demands t≥1, its lowest number of random numbers required to process *n* elements is 2kln(n/k).

Because the probability to select 2*k*-th element is 0.5, the average number of random numbers needed for this element is the same between Algorithm R and the optimal next selection method. Thus, up until 2*k*-th element, Algorithm R is expected to generate fewer random numbers than the optimal next selection method. To estimate the proportional difference of the random numbers needed in Algorithm R with respect to the optimal next selection method, we have for k≫1. Thus, Algorithm R generates 28% fewer random numbers than the best next selection method.


(3) $$\frac{k}{2k\ln\left(\frac{2k}{k}\right)}=\frac{1}{2\ln\left(2\right)}\approx 0.72.$$

### 5.6 Estimation of the number of random numbers needed in s-leaping

Because s-leaping uses Algorithm R to process the first 2*k* elements, it generates exactly *k* random numbers for the first 2*k* elements. After this point, s-leaping demands two random numbers per selection step, the lowest of the next selection method. Thus, the total number of random numbers generated in s-leaping to process *n* elements is expected to be


(4) $$\begin{array}{c} k+2\sum_{i=2k+1}^{n}\frac{k}{i}\approx k+2k\ln\left(\frac{n}{2k}\right)\\ =k+2k\ln\left(\frac{n}{k}\right)-2k\ln\left(2\right)\\ \approx 2k\ln\left(\frac{n}{k}\right)-0.39k. \end{array}$$

Because the optimal next selection method requires 2kln(n/k) to process *n* elements, s-leaping is expected to require 0.39*k* fewer random numbers than the optimal next selection method in total.

### 5.7 Runtime measure of the four reservoir-sampling methods

We implemented s-leaping and Algorithms R, L, and Z in C. To measure the CPU time of each method, we called the clock function right before and after the downsampling function call. We then converted the CPU clock count into the time unit of seconds to obtain the runtime. For each combination of *k* and *n*, the ratio of the average runtime of each next selection-based method to that of Algorithm R was sampled using 10 data points. To obtain the 95% confidence interval of the relative runtime, we assumed that runtime of each next selection method is independent of that of Algorithm R and used the second-order Taylor approximation method with zero covariance.

### 5.8 Computation of maximum D

For each combination of *k* and *n*, we performed downsampling 10 times, making the number of runs 120 for each method in total. In this downsampling, each reservoir element contained an index of a selected element, which is a number between 0 and n−1. The *k* selected numbers were then sorted and partitioned into 1000 bins to measure the empirical cumulative distribution at the permille resolution. We then computed the maximum D measure of the empirical cumulative distribution against the unit uniform distribution using the 1000 data points. The violin plots were generated from 40 maximum D samples for each method.

### 5.9 Leap-size factor in fadso

S-leaping has one tuning parameter, the leap-size factor ϵ. In fadso, we fixed this value to be 0.005. This value was determined so as to make the maximum discrepancy of the approximated selection probability reasonably small under a wide range of settings. The selection probability of the *i*-th element is k/i; hence, the maximum discrepancy between s-leaping and the exact reservoir sampling occurs when i=2k+s, with the relative difference being


(5) $$\begin{array}{c} \frac{k/(2k+0.5s)-k/(2k+s)}{k/(2k+s)}=\frac{2k+s}{2k+0.5s}-1\\ =\frac{2+\epsilon }{2+0.5\epsilon }-1\\ =\frac{0.5\epsilon }{2k+0.5\epsilon }. \end{array}$$

With ϵ=0.005, this upper limit becomes 0.00125.

### 5.10 Performance measurement of the FASTQ downsampling tools

The forward read FASTQ file of ERR174310 from the Illumina Platinum Sequencing project (study accession: PRJEB3246) was downloaded from European Nucleotide Archive. This FASTQ dataset contained 208M 200 bp read-pairs and to evaluate the performance of FASTQ downsampling tools, it was downsampled to have read counts ranging from 5 to 20 M with 5 M increment. For each downsampling size, five single-threaded runs were performed, and the CPU time and peak memory size from each run were measured using the GNU time program. The runtime was computed by adding the user CPU time and the system CPU time; the peak memory size was computed from the maximum resident set size.

### 5.11 Runtime environment information

All of the runs were carried out on an Intel Xeon Gold 6130 machine with 132 GB of RAM.

## Data Availability

The WGS sample from the Illumina Platinum genome project that we used to evaluate the performance of fadso is available with accession number ERR174310 from European Nucleotide Archive.

## Code availability

The C source code to run s-leaping, Algorithms R, L, and Z, as well as the fadso package is available at https://github.com/hkuwahara/sleaping. The fadso tool depends on the zlib library. And to compile the source and generate an executable, cmake is used.
